# Identification of TIMP2 as a Prognostic Biomarker and Its Correlation with Tumor Immune Microenvironment: A Comprehensive Pan-Cancer Analysis

**DOI:** 10.1155/2022/9133636

**Published:** 2022-10-18

**Authors:** Dan-Dan Wang, Wen-Xiu Xu, Wen-Quan Chen, Lei Li, Su-Jin Yang, Jian Zhang, Jin-Hai Tang

**Affiliations:** Department of General Surgery, The First Affiliated Hospital with Nanjing Medical University, 300 Guangzhou Road, Nanjing 210029, China

## Abstract

**Background:**

Tissue inhibitor of metalloproteinase-2 (TIMP2), an endogenous inhibitor of matrix metalloproteinases, has been disclosed to participate in the development and carcinogenesis of multiple malignancies. However, the prognosis of TIMP2 in different cancers and its correlation with tumor microenvironment and immunity have not been clarified.

**Methods:**

In this study, we conducted a comprehensive bioinformatics analysis to evaluate the prognostic and therapeutic value of TIMP2 in cancer patients by utilizing a series of databases, including Oncomine, GEPIA, cBioPortal, GeneMANIA, Metascape, and Sangerbox online tool. The expression of TIMP2 in different cancers was analyzed by Oncomine, TCGA, and GTEx databases, and mutation status of TIMP2 in cancers was then verified using the cBioPortal database. The protein-protein interaction (PPI) network of the TIMP family was exhibited by GeneMANIA. The prognosis of TIMP2 in cancers was performed though the GEPIA database and Cox regression. Additionally, the correlations between TIMP2 expression and immunity (immune cells, gene markers of immune cells, TMB, MSI, and neoantigen) were explored using Sangerbox online tool.

**Results:**

The transcriptional level of TIMP2 in most cancerous tissues was significantly elevated. Survival analysis revealed that an elevated expression of TIMP2 is associated with unfavorable survival outcome in multiple cancers. Enrichment analysis demonstrated the possible mechanisms of TIMPs and their associated genes mainly involved in pathways including extracellular matrix (ECM) regulators, degradation of ECM and ECM disassembly, and several other signaling pathways.

**Conclusions:**

Our findings systematically dissected that TIMP2 is a potential prognostic maker in various cancers and use the inhibitor of TIMP2, which may be an effective strategy for cancer therapy to improve the poor cancer survival and prognostic accuracy, but concrete mechanisms need to be validated by subsequent experiments.

## 1. Introduction

Cancer, a vicious disease, is the second leading cause of death, and the statistics are daunting globally [1]. Given the situation, the requirement for biomarker-matched molecularly targeted treatment for cancers shows the trend of increasing recognition. The investigation of novel and promising biomarkers as cancer mediators and therapeutic targets has now spanned multiple decades. In order to pinpoint novel biomarkers and to develop new interventions, we firstly and comprehensively delineated the expression spectrums and prognostic value of tissue inhibitor of metalloproteinases 2 (TIMP2) in diverse malignancies, which triggered fundamental cellular responses and was a vital player during tumorigenesis.

TIMPs are proteins approximately 21 kDa in molecular weight and consisting of 184–194 amino acids [2, 3]. They are dimers composed of an N-terminal domain and a smaller C-terminal domain binding to the MMP substrate [4]. Thus, the family of TIMPs (TIMP-1, 2, 3, 4) are able to mediate the degradation of MMPs and prominently appreciated as inhibitors of MMP activity [4, 5]. MMPs, also known as matrixins, primarily regulated the remodeling of the ECM components, which functions in many pathological conditions such as tumor cell invasion and metastasis, cell growth and differentiation, angiogenesis, and apoptosis [2, 6, 7]. TIMP2, ascribed to tissue inhibitors of metalloproteinase (TIMPs) family members, functioned as an endogenous inhibitor of matrix metalloproteinases (MMPs) and a homeostatic regulator at the interface between extracellular matrix (ECM) and cellular components [8, 9]. TIMP2, located on chromosome 17q25, has been indicated in the modulation of MMP-2 proteolytic activity via formation of a 1 : 1 stoichiometric suppressive complex with the enzyme [10, 11]. Tumor environment (TME) was coincident with increasing levels of active MMP expression, which was overwhelmed by TIMP2, resulting in tumor promoting functions [8]. TIMP2 showed that it exhibited a multiple interactions with components of the ECM by targeting several putative receptors, such as membrane-bound MMP146 [12, 13], integrin *α*3*β*15 [12], and insulin-like growth factor 1 receptor (IGFR1) [9]. These implicated that TIMP2 was involved in multiple different cancer-promoting processes, aiding discoveries in identifying therapeutic targets regarding the TIMP-metalloproteinase-substrate network.

Clinical cancer bioinformatics was emphasized as a crucial tool and emerging science, which might serve as a new paradigm for guiding cancer research. Recently, escalating online platforms for the mining, sharing, analysis, and integration of cancer data have come into existence. In this study, we had a sophisticated understanding of TIMP2 in pan cancer on basis of data-mining analysis from various databases, providing a theoretical basis for cancer diagnosis and prognosis. A preprint of our article has previously been published [14].

## 2. Materials and Methods

### 2.1. Oncomine Database

Oncomine (http://www.oncomine.org) is a free and public cancer microarray data for academic research community [15]. The relative mRNA expression of TIMP2 in various cancer tissues compared with the normal tissues is analyzed by Oncomine. The thresholds are defined at *p* vaule≤1*E*-4, fold change ≥ 2, and gene rank top 10%.

### 2.2. cBioPortal Database

The cBioCancer Genomics Portal (cBioPortal database, http://cbioportal.org) is a newly developed interactive, open-access web server for the exploration of numerous cancer genomics datasets, based on the data retrieved from the TCGA database [16]. Analysis of the genomic alterations of TIMP2 included copy number amplification, deep deletion, missense mutation with uncharted significance, and mRNA upregulation. 32 studies (10967 samples) in *Pancancer studies* module were selected.

### 2.3. GEPIA Database

The GEPIA (Gene Expression Profiling Interactive Analysis) database (http://gepia.cancer-pku.cn/index.html) is an open-access web resource for analyzing the RNA sequencing expression data from the TCGA and the Genotype-Tissue Expression (GTEx) database and provides customizable functions including differential expression analysis, correlation analysis, and survival analysis [17] . In the current study, we mainly used the GEPIA database to get the overall survival (OS) and DFS data of TIMP2 of high level of TIMP2 patients and low levels of TIMP2 patients.

### 2.4. GeneMANIA Database

The GeneMANIA (https://www.genemania.org/) was adopted to predict the potential functions of TIMP2. GeneMANIA produced a series of genes with similar functions to TIMP2 and exhibited a gene-gene interaction network to expound relationships between TIMP2 and its associated genes. In this study, we constructed this interactive functional-association network for TIMP2 in terms of genetic interactions, coexpression, colocalization, physical interactions, predictions, and protein domain similarity [18].

### 2.5. Relationship between TIMP2 Expression and Immunity

Cancer progression is an intricate process controlled by a series of factors that coordinate the crosstalk between immune components of TME and the tumor cells. Knowledge of the sophisticated interplay between tumor and immunity could aid in formulating novel combination treatments to conquer tumor immune evasion mechanisms and direct immunotherapy decision-making. Attuned with these facts, we explored the relationship between the level of TIMP2 expression and immunity by using Sangerbox online tool, including infiltrating immune cells, gene markers of immune cells, tumor mutational burden (TMB), microsatellite instability (MSI), and neoantigen.

### 2.6. Functional and Pathway Enrichment Analysis

Functional and pathway enrichment analysis of TIMP family members and coexpressed genes was next performed using Metascape. Metascape website (http://metascape.org) is a friendly and well-maintained gene-list analysis online tool for gene analysis and annotation, which integrated analysis tools and biological information to offer a systematic annotation [19]. The Molecular Complex Detection (MCODE) algorithm was employed to screen the densely connected modules of PPI network. Gene Ontology (GO) terms for biological process, cellular component, and molecular function categories were enriched based on the Metascape online tool.

### 2.7. Single-Cell Functional Analysis

The functional state of TIMP2 in diverse cancer types was assessed by CancerSEA (http://biocc.hrbmu.edu.cn/CancerSEA/). CancerSEA, a comprehensive database aimed at delineating a cancer single-cell functional state atlas, covers 14 functional states of 41,900 cancer single cells from 25 tumor types. These functional states include stemness, invasion, metastasis, proliferation, EMT, angiogenesis, apoptosis, cell cycle, differentiation, DNA damage, DNA repair, hypoxia, inflammation, and quiescence [20].

### 2.8. Statistical Analysis

The expression data from the Oncomine database is analyzed by Student's *t*-test. Transcripts per million (TPM) serve as a measurement of the proportion of transcripts in the pool of RNA. The expression level of TIMP2 is showed with log2 TPM. The prognostic values of high- and low-expression groups were evaluated according to the hazard ratio (HR), 95% confidence interval (CI), and log-rank *P* values. *P* value <0.05 indicated statistically significant differences.

## 3. Results

### 3.1. The Expression and Mutation Profiling of TIMP2 in Different Cancer Types

**Figure 1:**
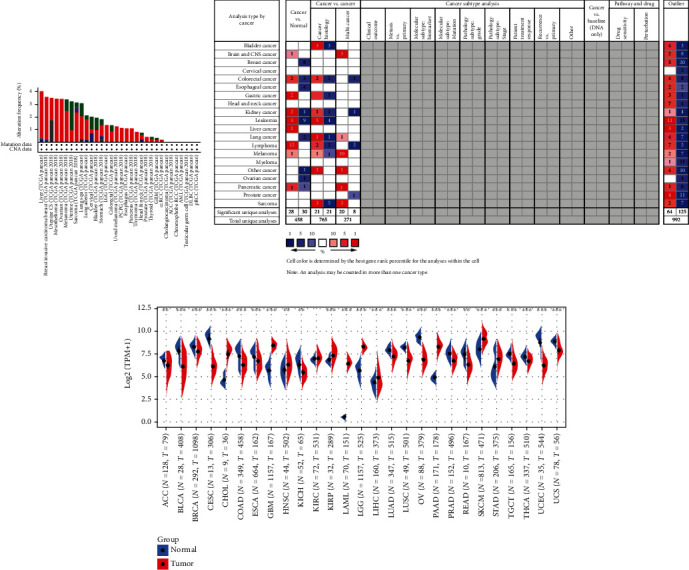
Genomic alterations and mRNA expression landscape of TIMP2 in different types of cancers. (a) The genetic alteration type and frequency of TIMP2 in diverse malignancies. The results are displayed as a histogram of the alteration frequencies of TIMP2 across cancer studies. The genetic alteration type and frequency included amplification (red), deep deletions (blue), mutation (green), fusion (purple), and multiple alterations (grey). Color images are available online. (b) The Oncomine database showed high or low expression of TIMP2 in various cancer tissues compared with normal tissues. Red and blue stand for the numbers of datasets with statistically significant (*P* < 0.05) increased and decreased levels of PTPN family genes. (c) Transcripts per million (TPM) of TIMP2 in different cancer types from TCGA and GTEx data. The red fusiformis represents tumor tissue, and the blue fusiformis represents normal tissue. T: tumor; N: normal; n: number; X axis: number of tumor and normal samples; Y axis: transcript per million (log2 (TPM+ 1)). ^∗^*P* < 0.05, ^∗∗^*P* < 0.01, and ^∗∗∗^*P* < 0.001.

Cancer is a disease of the genome and develops as one end-product of accumulating somatic mutation [21, 22]. Remarkable advances in next-generation sequencer (NGS) and computational technology dealing with massive data make it available to synthetically analyze cancer genome profiles at clinical and research levels [22]. Thus, our aim was to explore the genomic mutation profiling of TIMP2 in pan cancer, regarding the analysis of TIMP2, which was exhibited by the cBioPortal database. The genetic alteration characterization of TIMP2 showed that its amplification was one of the most important single factors for alteration in liver cancer, BRCA (breast invasive carcinoma), mesothelioma, sarcoma, lung adenocarcinoma, LGG, CRC, uveal melanoma, PCPG, esophagus cancer, pancreas cancer, thyroid cancer, GBM, and ccRCC. Besides, TIMP2 mutation frequencies are the highest in liver cancer, BRCA, and mesothelioma (Figure 1(a)). The Oncomine database showed that TIMP2 mRNA levels were significantly upregulated in nine cancer datasets, especially lymphoma (15 reported). Meanwhile, leukemia was the most down-expression cancer type (9 reported). Additionally, we visualized the expression of TIMP2 genes in various cancer tissues and adjacent tissues in Figure 1(c), and the higher TPM levels of TIMP2 in multiple cancers were observed (*P* < 0.05). Data extracted from the TCGA database revealed that the TIMP2 expression was notably higher in 10 tumor types compared to matched TCGA normal tissues and GTEx data, including CHOL, GBM, HNSC, KIRP, LAML, LGG, LIHC, PAAD, SKCM, and STAD (Figure 1(c)).

### 3.2. The Prognostic Significance of TIMP2 Expression in Different Cancer Types

**Figure 2:**
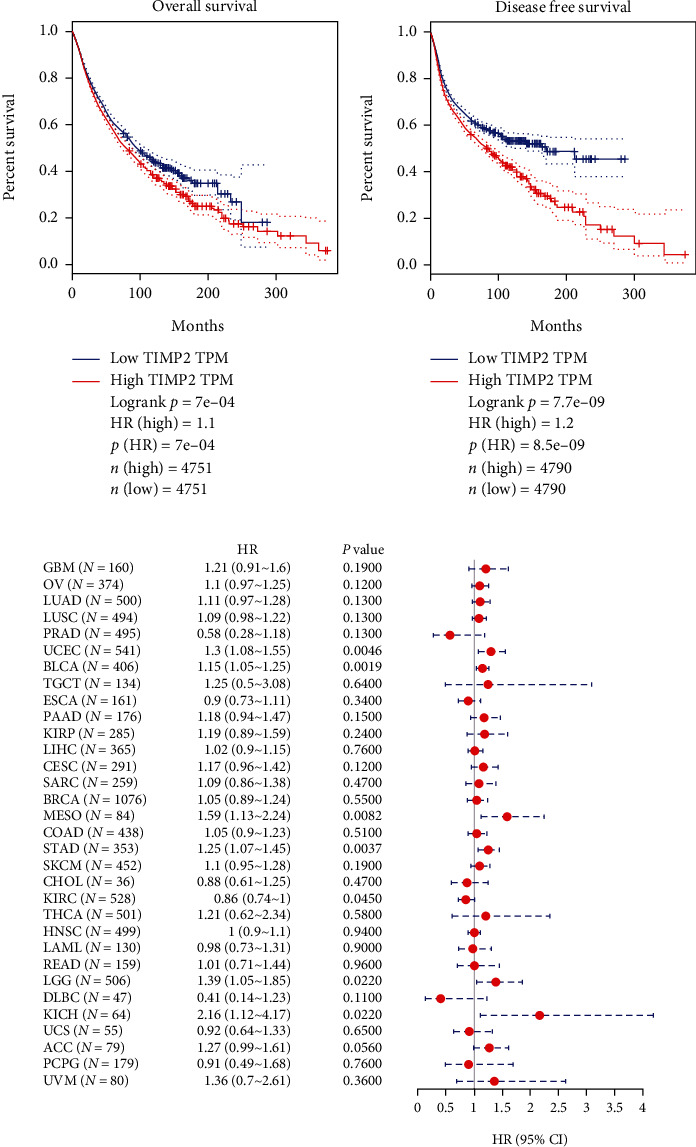
The prognostic value of the TIMP2 mRNA expression in cancer patients. (a) OS, (b) DFS, and (c) forest plot disclosed the result of survival analysis in pan cancer, 95% (CI), and *P* value of TIMP2 in each individual cancer. Red dots represent HR. Abbreviation: HR: hazard ratio; CI: confidence interval; OS: overall survival; DFS: disease-free survival. The *P* values were calculated using the log-rank test. ^∗^*P* < 0.05.

Kaplan Meier curves displayed elevated expression of TIMP2 was an unfavorable prognostic factor for cancer patients, including OS (overall survival, Figure 2(a)) and DFS (disease-free survival, Figure 2(b)) prognosis. As shown in Figure 2(c), the high mRNA expression of TIMP2 predicted worse survival for UCEC (HR = 1.3, 95% CI: 1.08-1.55, *P* = 0.0046), BLCA (HR = 1.15, 95% CI: 1.05-1.25, *P* = 0.0019), MESO (HR = 1.59, 95% CI: 1.13-2.24, *P* = 0.0082), STAD (HR = 1.25, 95% CI: 1.07-1.45, *P* = 0.0037), LGG (HR = 1.39, 95% CI: 1.05-1.85, *P* = 0.022), and KICH (HR = 2.16, 95% CI: 1.12-4.17, *P* = 0.022), respectively.

### 3.3. The Correlation between TIMP2 Expression and Immune Infiltrates

**Figure 3:**
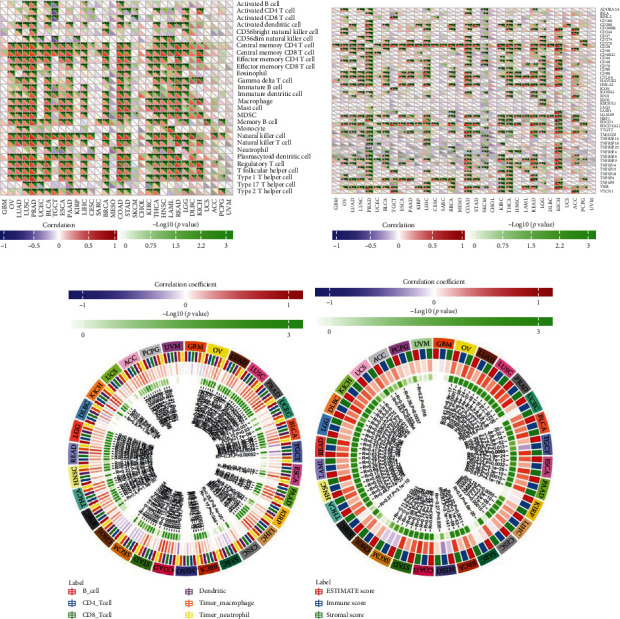
Relationship between TIMP2 expression and immune infiltration level in pan cancer. (a) Relationship between TIMP2 expression and infiltration level of 22 immune cell subtypes. (b) Relationship between TIMP2 expression and immune marker sets. (c) Relationship between TIMP2 expression and infiltration level of the most common immune cells, including B cell, CD8+ T cell, CD4+ T cell, macrophage, neutrophil, and dendritic cell. (d) Relationship between the estimated proportion of immune and stromal and TIMP2 expression in pan cancer, and analysis was used by ImmuneScore, StromalScore, and ESTIMATEScore.

**Figure 4:**
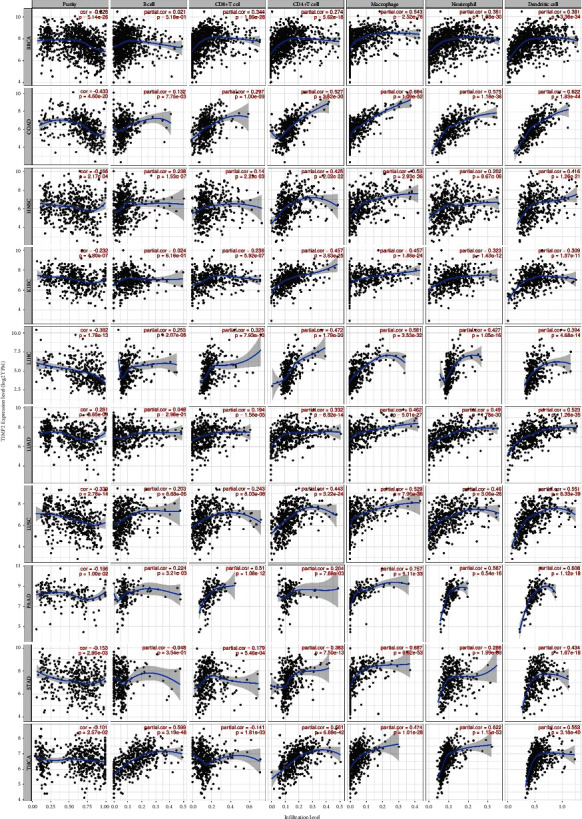
Correlation of the TIMP2 expression with immune infiltration levels of B cells, CD8 + T cells, CD4 + T cells, macrophages, neutrophils, and dendritic cells in BRCA, COAD, HNSC, KIRC, LIHC, LUAD, LUSC, PAAD, STAD, and THCA.

When analyzing the association between TIMP2 expression and immune subtypes, it was found that the expression of TIMP2 was most positively associated with central memory CD4+ T cell, central memory CD8+ T cell, effector memory CD4+ T cell, effector memory CD8+ T cell, gamma delta T cell, immature dendritic cell, mast cell, MDSC, memory B cell, natural killer cell, natural killer T cell, plasmacytoid dendritic cell, regulatory T cell, T follicular helper cell, and type 1 T helper cell. Furthermore, TIMP2 was most positively associated with major immune cells in OV, LUAD, LUSC, PARD, BLCA, ESCA, PAAD, LIHC, BRCA, COAD, STAD, THCA, READ, and LGG (Figure 3(a)). With regards to gene markers of immune cells, the expression of TIMP2 was found to positively correlate with CD276. PRAD, COAD, THCA, and KICH were top four tumors which had the most gene markers of immune cells positively associated with the TIMP2 expression (Figure 3(b)). Analysis of the relationship between TIMP2 expression and six common immune cells revealed that the expression of TIMP2 positively correlated with COAD, LIHC, PRAD, LUAD, OV, ACC, LGG, READ, and THCA (Figure 3(c)). In addition, our study found that the TIMP2 expression was positively correlated with ImmuneScore, StromalScore, and ESTIMATEScore in THCA, HNSC, LAML, READ, LGG, DLBC, KICH, OV, LUAD, LUSC, PRAD, BLCA, ESCA, TGCT, and PAAD (Figure 3(d)). Furthermore, we evaluated the association of TIMP2 with levels of immune cell infiltration in cancers. As shown in Figure 4, TIMP2 was significantly correlated with B cells, CD8 + T cells, CD4 + T cells, macrophages, neutrophils, and dendritic cells in multiple cancers, including BRCA, COAD, HNSC, KIRC, LIHC, LUAD, LUSC, PAAD, STAD, and THCA. These results suggested that the TIMP2 expression might be involved in regulating the aforementioned immune molecules and play a vital role in immune microenvironment.

### 3.4. Relationship between TIMP2 Expression and TMB, MSI, and Neoantigen

**Figure 5:**
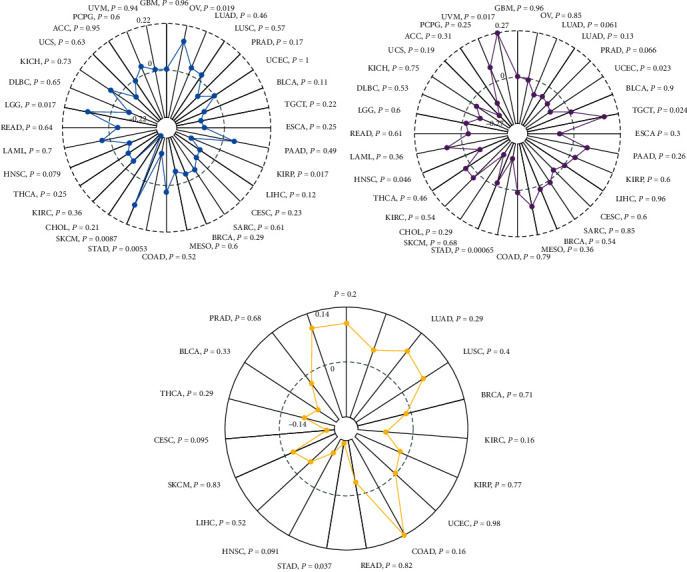
Radar maps of relationship between TIMP2 expression and (a) TMB, (b) MSI, and (c) neoantigen.

TMB is defined as the number of somatic mutations detected on NGS per megabase (mb) [23, 24]. As measured by immunohistochemistry, high TMB is an emerging biomarker of predicting the response to immune checkpoint inhibitors [25]. Across tumor diagnoses, patients with high TMB might be an optimal subgroup for ICI therapy and have a higher likelihood of immunotherapy [24, 26]. More broadly, neoantigens arise from tumor-specific mutations that differ from wild-type antigens, which is a major factor in the activity of clinical immunotherapies and may guide application of immunotherapies [27] [28]. These observations indicated that TMB, MSI, and neoantigen might form biomarkers in the immune response to cancer patients and provide the progress of novel therapeutic approaches with an incentive. In addition, TIMP2 was positively correlated with TMB in OV, LGG, and SKCM and negatively correlated with TMB in STAD and KIRP (Figure 5(a)). TIMP2 was positively correlated with MSI in UVM and TGCT and negatively correlated with MSI in HNSC, STAD, and UCEC (Figure 5(b)). TIMP2 was negatively correlated with neoantigen in with MSI in STAD (Figure 5(c)).

### 3.5. Functional Annotation of Coexpression Gene Network of TIMP2

**Figure 6:**
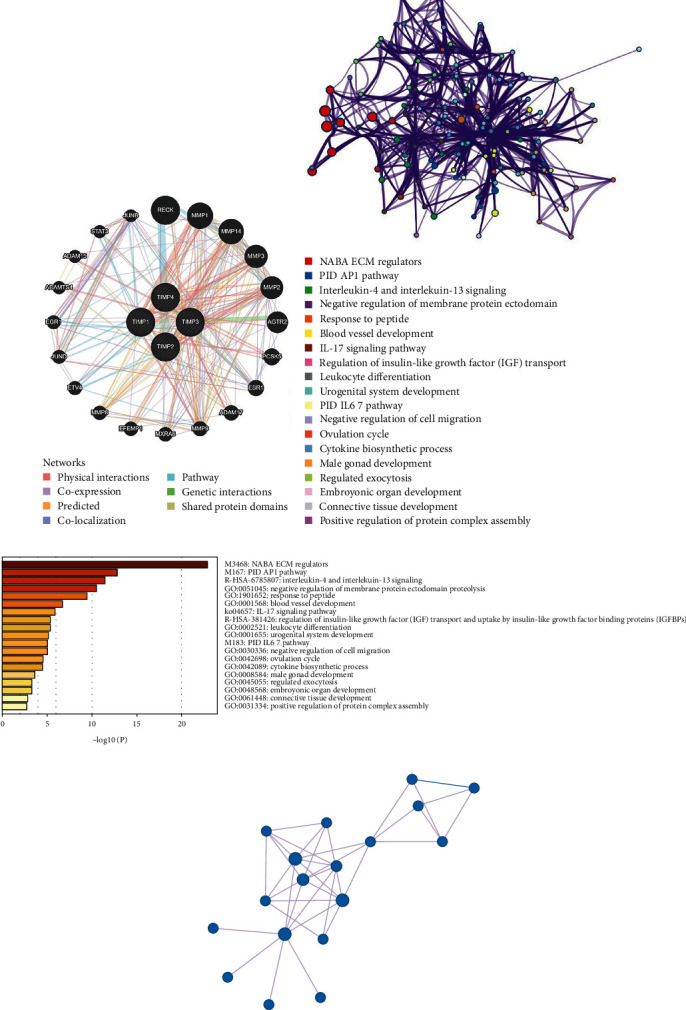
Functional role of TIMP2 and potential mechanisms in cancers. (a) Gene-gene interaction network analysis of TIMP family members obtained from the GeneMANIA database. Each node represented a gene. The node size indicated the strength of interactions. The internode connection lines indicated the types of gene–gene interactions, and the line color indicated the types of interactions. (b) Network of GO enriched terms colored by clusters. (c) The bar plot of GO enriched terms of the genes coexpressed with TIMP2 colored by *P* value. (d) Most significant MCODE components form the PPI network. GO: Gene Ontology.

The TIMP family (TIMP-1, 2, 3, 4), a class of transcription factors, has four members, which are roughly 40% identical in amino acid sequence, and TIMP2 and TIMP4 share most similarities [3]. As shown in Figure 6(a), 20 genes showed the greatest association with TIMPs in the gene interaction network, including RECK, MMP1, MMP14, MMP3, MMP2, AGTR2, PCSK5, ESR1, ADAM17, MMP9, MXRA8, EFEMP1, MMP8, ETV4, JUND, EGR1, ADAMTS4, ADAM15, STAT3, and JUNB. Further functional analysis revealed that the top six pathways related to these genes were NABA ECM regulators (log*P* = −22.8947, *z* − score = 30.68476), PID AP1 pathway (log*P* = −19.2461, *z* − score = 37.29148), IL-4 (interleukin-4) and IL-13 (interleukin-13) signaling (log*P* = −19.1111, *z* − score = 31.49618), negative regulation of membrane protein ectodomain proteolysis (log*P* = −17.967, *z* − score = 47.44123), response to peptide (log*P* = −15.867, *z* − score = 16.87997), and blood vessel development (Figures 6(b) and 6(c)). Moreover, it was also related to the metabolism of insulin, glucose, and fat and cell surface receptor signaling pathways which regulate immune response. The top 3 most relevant MCODE modules were NABA ECM regulators, degradation of the extracellular matrix, and extracellular matrix disassembly (Figure 6(d)).

### 3.6. Functional States of TIMP2 across Various Cancer Types

**Figure 7:**
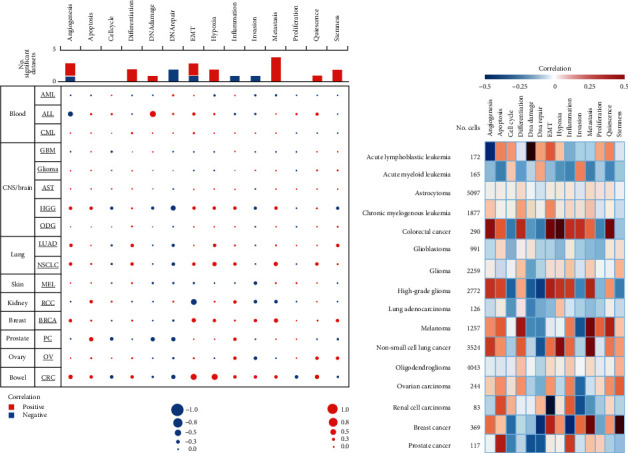
Single-cell sequencing analysis of TIMP2 using the CancerSEA database. (a, b) The functional state of TIMP2 across diverse types of cancers. The red pots represented that TIMP2 was positively correlated with the functional state while the blue pots represented that TIMP2 was negatively correlated with the functional state.

To get a better understanding of the enigmatic and sophisticated role of the TIMP2 expression in cancer, we explored the functional state of TIMP2 across various cancer types based on the CancerSEA database. TIMP2 has been explored at the single-cell resolution in sixteen types of cancers (Figures 7(a) and 7(b)). TIMP2 was positively correlated with apoptosis (*R* = 0.501, *P* = 0.001) and negatively correlated with angiogenesis (*R* = −0.388, *P* = 0.015) in acute lymphoblastic leukemia. TIMP2 was positively correlated with metastasis in multiple cancers, including high-grade glioma (*R* = 0.289, *P* < 0.001), non-small-cell lung cancer (*R* = 0.393, *P* < 0.001), and BCa (*R* = 0.403, *P* < 0.001). TIMP2 was positively correlated with angiogenesis (*R* = 0.349, *P* = 0.032), EMT (*R* = 0.404, *P* = 0.012), hypoxia (*R* = 0.441, *P* = 0.006), and in quiescence (*R* = 0.398, *P* = 0.013). However, TIMP2 was not significantly correlated with any of the 14 functional states in glioma.

## 4. Discussion

TIMPs firmly participated in the development and process of the majority of cancer hallmarks and may serve as promising biomarkers for cancer prognosis in patient body fluids [3]. A systematic and comprehensive understanding of the TIMP-metalloproteinase-substrate network will aid in MMP inhibitor design for therapy. As numerous studies delineated protease-independent TIMP function and protease biology was inherent to various human cancers, advances made in comprehending these versatile metalloproteinase inhibitors could help us defeat cancers. Future efforts will align animal model systems with changes in TIMPs in patients, will pinpoint therapeutic targets within the TIMP-metalloproteinase-substrate network, and will use TIMPs in liquid biopsy samples as biomarkers for cancer prognosis. Among the family of TIMPs, Wang et al. disclosed that TIMP2 participated in the regulation of cell adhesion, angiogenesis, and epithelial-to-mesenchymal transition (EMT) and interacted with multiple integrin pathways [29]. Upregulated TIMP2 expression level in cancer tissues probably played a crucial part in the occurrence of cancers. Additionally, TIMP2 probably exerted their functions in many aspects of tumorigenesis through ECM regulators, degradation of ECM, and ECM disassembly.

Cancer immunotherapy has shown substantial and validated therapeutic effects in patients with cancer, including ICI and adoptive cell therapy, manipulating the immune system to discern and assault cancer cells [30, 31]. As introduced previously, TIMP2 was related to TMB, MSI, and neoantigen in varying degrees, providing a theoretical basis for directing patient-specific cancer immunotherapy optimizing clinical benefit of current immunotherapy.

Altogether, our study was conducted using diverse public databases and displayed the expression and clinical significance of TIMP2 in cancers. However, our research has several limitations. Sample numbers of each cancer varied greatly, which may lead to a reduction in the reliability and accuracy of analyses for those cancers with a relatively small sample size. In addition, our analysis failed to account for differences in clinical and sociodemographic characteristics of the individuals. The biological interactions and detailed mechanisms involved need further investigation and experimental confirmation, which will be conducted in future researches. We hope our study may be helpful to potential prognostic markers for the improvement of cancer survival and prognostic accuracy in the future.

## 5. Conclusions

Comprehensive understanding of the TIMP2 may have guiding significance for the prognostic judgments, early diagnosis, and targeted therapy of in cancer patients. Based on our study, further discovery of the systematic molecular mechanisms that how TIMP2 interacted with different signaling and other molecules or leads to different prognosis of cancer patients can pave the way for more effective tumor diagnosis and serve as a genetic treatment target. Therefore, we hypothesize that TIMP2 is a versatile candidate as a novel biomarker and therapeutic target for cancers.

## Data Availability

The data used to support the findings of this study are available from the corresponding author upon request.

## Abbreviations

ACC: Adrenocortical carcinoma
BLCA: Bladder urothelial carcinoma
BRCA: Breast invasive carcinoma
COAD: Colon adenocarcinoma
DLBC: Lymphoid neoplasm diffuse large B-cell lymphoma
ESCA: Esophageal carcinoma
GBM: Glioblastoma multiforme
HNSC: Head and neck squamous cell carcinoma
KICH: Kidney chromophobe
KIRC: Kidney renal clear cell carcinoma
KIRP: Kidney renal papillary cell carcinoma
LAML: Acute myeloid leukemia
LGG: Brain lower grade glioma
LIHC: Liver hepatocellular carcinoma
LUAD: Lung adenocarcinoma
LUSC: Lung squamous cell carcinoma
OV: Ovarian serous cystadenocarcinoma
PAAD: Pancreatic adenocarcinoma
PRAD: Prostate adenocarcinoma
READ: Rectum adenocarcinoma
SKCM: Skin cutaneous melanoma
STAD: Stomach adenocarcinoma
TGCT: Testicular germ cell tumors
THCA: Thyroid carcinoma
THYM: Thymoma
UCEC: Uterine corpus endometrial carcinoma
UCS: Uterine carcinosarcoma
TIMP2: Metalloproteinase 2
MMPs: Matrix metalloproteinases
ECM: Extracellular matrix
TME: Tumor environment
IGFR1: Insulin-like growth factor 1 receptor
NGS: Next-generation sequencer
EMT: Epithelial-to-mesenchymal transition
IL-4: Interleukin-4
IL-13: Interleukin-13
OS: Overall survival
DFS: Disease-free survival.
